# Case report: cerebral venous sinus thrombosis and pulmonary embolism as the initial presentation in a child with asymptomatic primary nephrotic syndrome

**DOI:** 10.3389/fped.2023.1169116

**Published:** 2023-05-05

**Authors:** Qinhui Wang, Yaru Cui, Ping Liang, Chuan Wang, Kaiyu Zhou, Fan Ma, Hongyu Duan

**Affiliations:** ^1^ Department of Pediatrics, West China Second University Hospital, Sichuan University, Chengdu, China; ^2^ The Cardiac Development and Early Intervention Unit, West China Institute of Women and Children’s Health, West China Second University Hospital, Sichuan University, Chengdu, China; ^3^ West China Medical School of Sichuan University, Chengdu, China; ^4^ Key Laboratory of Birth Defects and Related Diseases of Women and Children of MOE, Department of Pediatrics, West China Second University Hospital, Sichuan University, Chengdu, China; ^5^ Key Laboratory of Development and Diseases of Women and Children of Sichuan Province, West China Second University Hospital, Sichuan University, Chengdu, China

**Keywords:** cerebral venous sinus thrombosis, pulmonary embolism, nephrotic syndrome, children, case report

## Abstract

**Background:**

Cerebral venous sinus thrombosis (CVST) is rare, but potentially life-threatening. The clinical course definitely become more unpredictable and fatal in patients complicated by pulmonary embolism (PE). Nephrotic syndrome (NS) is an uncommon etiology of CVST. Concurrence of CVST and PE at the initial onset of NS is extremely unusual and rarely reported. Considering that edema might be absent in NS individuals, thromboembolic events probably become unrecognized, thereby causing a missed or delayed diagnosis and poor outcome. Herein, we described an extraordinary case of an adolescent boy presenting with both CVST and PE initially just within 5 days of disease onset, who was ultimately diagnosed with asymptomatic NS, aiming to emphasize a high index of suspicion of these diseases in patients with conditions of hypercoagulability.

**Case presentation:**

A 13-year-old male child presented acutely with dizziness, fever and dyspnea, with signs of shock but undetected edema. Initial laboratory investigations revealed hypoalbuminemia, typical images of pneumonia, and normal radiographic findings on non-enhanced computed tomography of head. Despite evidence of hypoalbuminemia and neurological symptoms, the child was still misdiagnosed as pneumonia. His dyspnea and period of headache deteriorated even if hemodynamic stability and undetected fever after initial therapy. The delayed urinalysis and 24-h urine examination both showed massive proteinuria. A computed tomography angiography of chest along with cranial magnetic resonance imaging/magnetic resonance venography were subsequently performed, consistent with the imaging features of PE and CVST, respectively. The diagnosis of asymptomatic primary NS complicated by PE and CVST was ultimately confirmed. The patient received corticosteroids and antithrombotic therapy with satisfactory results.

**Conclusion:**

A persistent clinical suspicion of CVST should be borne in mind in patients with a sudden, new or worsening headache, specifically among those with prothrombotic conditions. NS should always be considered in the differential diagnosis of risk factors for CVST, even in absence of edema. Since CVST and PE can be present simultaneously at extraordinary early-onset of NS, early radiological diagnosis is clinically substantial to proper management and satisfactory long-term outcomes.

## Introduction

Cerebral venous sinus thrombosis (CVST) is a rare neurological condition characterized by venous thromboembolism (VTE) in cerebral venous or dural sinuses. It occurs in approximately 0.67 per 100,000 children with a male predominance under the age of 18 years (1, 2). CVST can present with a multitude of nonspecific signs and symptoms (e.g., headache, vomiting, seizure, lack of consciousness, and focal neurological deficits, et al.), making it difficult to distinguish from other neurological diseases (3). The most severe cases can have a rapid neurological deterioration, 15%–20% of whom have different degrees of permanent disability or die (4). Of note, on the ground that approximately 5% of CVST patients can be complicated by severe comorbidity such as pulmonary embolism (PE) (5), the clinical course definitely becomes more unpredictable and fatal in such situation. Therefore, early and adequate recognition of the life-threatening disease is clinically substantial to proper management and satisfactory long-term outcomes.

Like any thrombosis, CVST has a multifactorial etiology. The most common risk factors are diverse and differ from those observed in adults, which have been frequently addressed as head/neck infections (36.67%–42.9%), inherited thrombophilia (26.4%, e.g., factor V Leiden mutations, protein C/S deficiency and hyperhomocysteinemia), head trauma (11.67%), malignancies (8.33%–9.4%), and a central venous catheter (6.9%) (6–9). Specifically, nephrotic syndrome (NS), featuring with acquired hypercoagulability, only represents a rare underlying etiology for the occurrence of CVST, with a proportion ranging from 1.67% to 3.8% (6, 7, 9, 10). Among the patients suffering from NS, VTE is the most frequently thromboembolic complication. The resulting deep venous thrombosis (DVT), renal vein thrombosis (RVT) and PE account for more than 75% of all the events (11). Despite the unknown exact incidence in children, CVST is believed to be an uncommon or possibly underdiagnosed complication of NS (12). Furthermore, irrespective of etiology, the rate of PE has been estimated to be 1.4% in patients with CVST (13). Occurrence of CVST and PE with evidence of NS is extremely unusual and rarely reported (14). Moreover, as for the time point of occurrence of VTE in patients with NS, it occurs prevalently as part of the disease course or as a consequence of the pharmacological treatment, more frequently in relapsing NS or steroid resistant NS (15–17). The median time from NS diagnosis to the first thromboembolism is reported to be 49 days (17, 18). To our knowledge, no literature is available so far reporting CVST and PE as the initial manifestations of NS. Notably, considering that edema might be absent in a small proportion of affected individuals, NS is likely to become an unrecognized culprit in cases with combined CVST and PE, thereby causing a missed or delayed diagnosis and poor outcome. Herein, we described an extraordinary case of an adolescent boy presenting with both CVST and PE initially just within 5 days of disease onset, and was ultimately found to have an otherwise asymptomatic and corticosteroid-responsive NS.

## Case descriptions

A 13-year-old male child was referred to local hospital due to 5 days of dizziness, and 2 days of fever and dyspnea. Except for a history of neurodermatitis cured by antihistamines, the patient had normal nutritional status without other previous underlying diseases, head injury and drug exposures. Additionally, routine childhood vaccines had been implemented in this patient. No local epidemiology of communicable diseases had been reported in the area where the patient resided. On physical examination, the children exhibited nasal flaring with respiratory rates of 31 breaths/minute, in absence of cyanosis, pallor, retractions, crackles, heart murmurs, hepatosplenomegaly, or extremity edema. The chest x-ray revealed streaky opacities in both lungs. Review of the rest documents suggested hypoalbuminemia (2.1 g/dl), along with normal results of cerebrospinal fluid (CSF) analysis, non-enhanced computed tomography (CT) scan of head, aminotransferase, and renal function. As being supposed to be secondary to acute infections, no further effort was directed toward defining the underlying causes of hypoalbuminemia. The patient was initially diagnosed as pneumonia, and antibiotic treatment (oxacillin, 100 mg/kg per day) was administered. His condition deteriorated within 2 days of hospitalization, presenting with a progressive course of breathlessness, lethargy, and hemodynamic instability, along with elevated levels of leukocyte. Thus, the patient was directly transferred to the pediatric intensive care unit (PICU) in our hospital in view of critical illness. On arrival, he exhibited altered mental status, sunken eyes, dehydrated skin, cold extremities, and photoreactive pupil with hypothermia (35.7°C), tachypnea (35 breaths/minute), tachycardia (127 beats/min), hypotension (82/60 mmHg), and percutaneous oxygen saturation of 95% via a nasal cannula. Other than rough respiratory sounds, the rest of his physical examinations were unremarkable.

**Figure 1:**
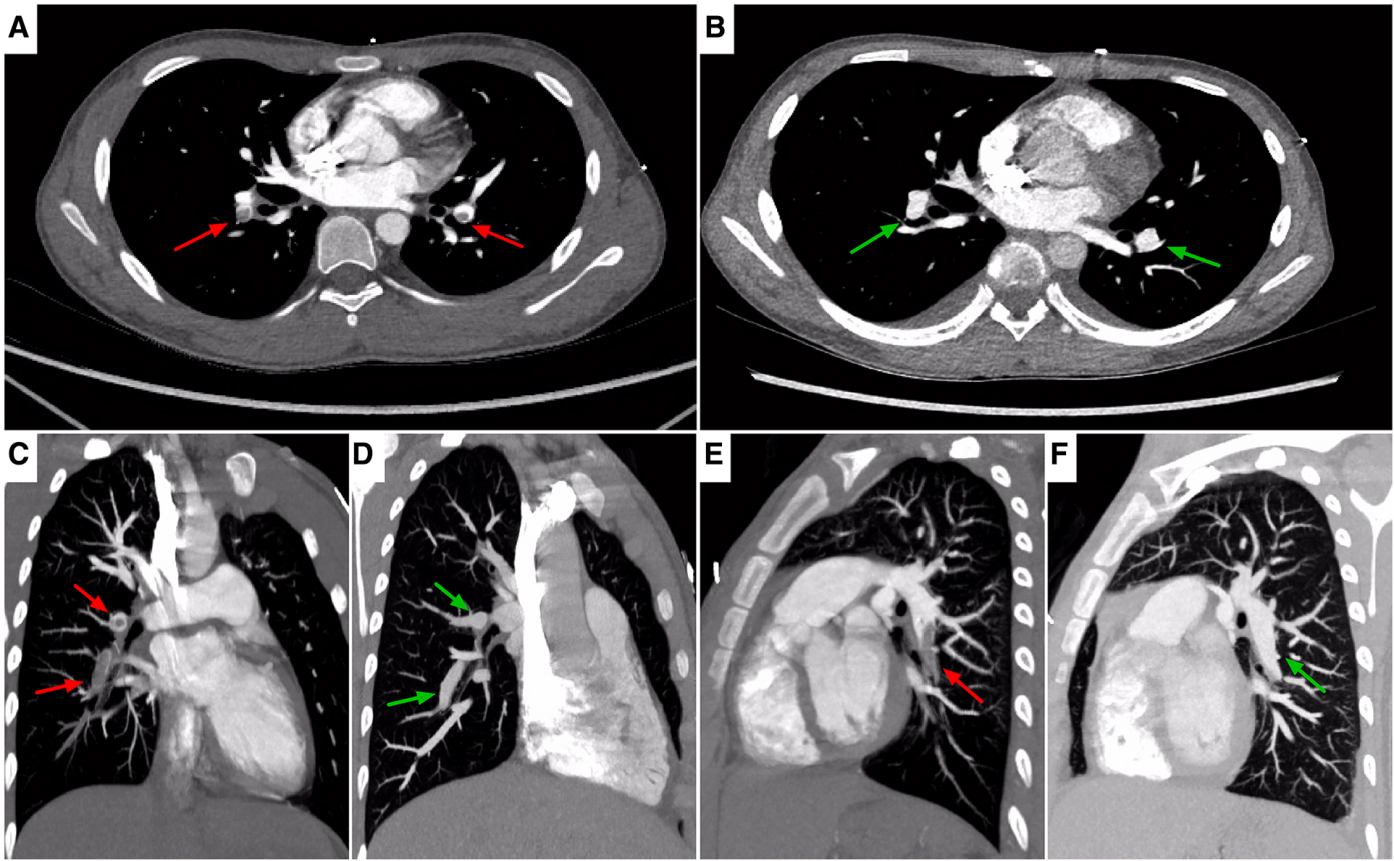
Chest CTA, axial (**A,B**), sagittal (**C–F**) images, showing locations of PE. On admission (**A,C,E**), images showing non-enhancing partial filling defects in bilateral pulmonary arteries extending into descending branches (red arrows). On the 6th day of antithrombotic therapy (**B,D,F**), images showing recanalization of the thrombosed vessels (green arrows). CTA, computed tomography angiography; PE, pulmonary embolism.

**Figure 2:**
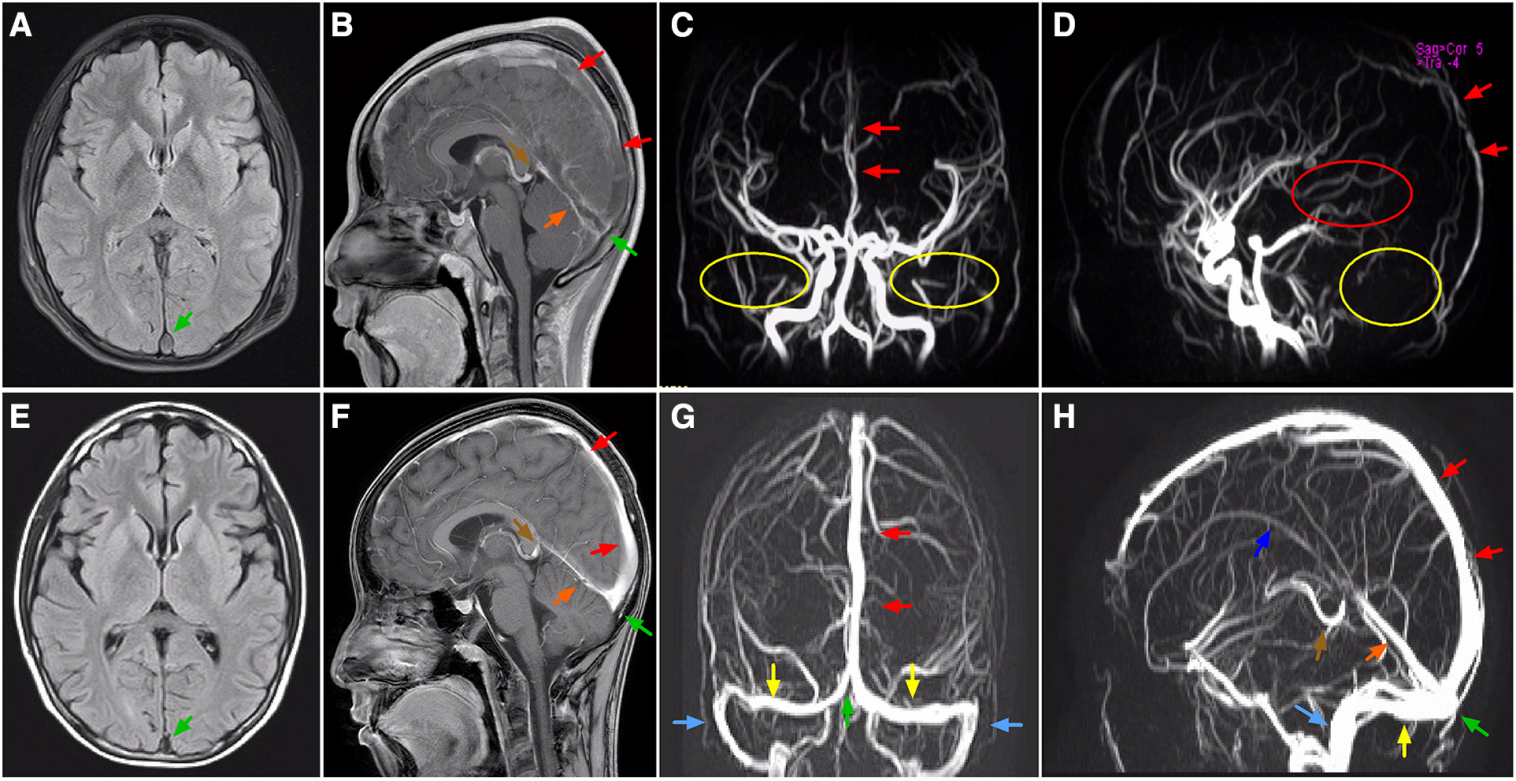
MRI and MRV, axial (**A,E**), sagittal (**B,F,D,H**) coronal (**C,G**) images, showing locations of CVST. On admission, T2-FLAIR MRI (**A**) and enhanced MRI (**B**), and MRV (**C,D**) images showing complete (partial) filling defect in superior sagittal sinus, straight sinus, vein of Galen, and torcula (arrows), together with absence of flow related signals where the sigmoid sinus (yellow circle), bilateral transverse sinus (yellow circle), and inferior sagittal sinus (red circle) normally existed. On the 20th day of antithrombotic therapy, T2-FLAIR MRI (**E**) and enhanced MRI (**F**), and MRV (**G,H**) images showing recanalization of the thrombosed vessels (arrows). Green arrow, torcula; red arrow, superior sagittal sinus; orange arrow, straight sinus; brown arrow, vein of Galen; yellow arrow, transverse sinus; blue arrow, sigmoid sinus; purple arrow, inferior sagittal sinus. MRI, magnetic resonance imaging; MRV, magnetic resonance venography; CVST, cerebral venous sinus thrombosis; FLAIR, fluid attenuated inversion recovery.

Initial laboratory investigations performed on day 1 of hospitalization had revealed increased leukocyte (21,600/μl) with 84.3% neutrophils and 9.4% lymphocytes, red blood cell (RBC) count (4.14 × 10^6^/μl), hemoglobin (11.3 g/dl), hematocrit (33.9%), procalcitonin (0.8 ng/ml), and C-reactive protein (CRP) (1.89 mg/dl) with normal platelets (39.4 × 10^4^/μl). Results of renal function, electrolytes, and blood gas analysis were within normal ranges. Decreased antithrombin III level (44%) combined with elevation of fibrin degradation products (FDP, 45.8 μg/ml), D-dimer (DDI, 7.9 μg/ml) and fibrinogen (553 mg/dl), were identified. Moreover, Serum albumin was low at 2.4 g/dl (Table 1). Chlamydia/mycoplasma antibody, PPD test, myocardial troponin I, complement level, antistreptolysin O (ASO) titer, RT-PCR for SARS-CoV-2, blood culture, and echocardiography demonstrated normal findings. Urine examination was not performed because of anuria, while subsequent ultrasound did not revealed structural anatomic anomalies, hydronephrosis or obstructive uropathy. Repeat chest x-ray displayed bibasilar nodular opacities with consolidation. The patient was diagnosed as pneumonia combined with shock. Right internal jugular catheterization was placed under ultrasound guidance. The patient was then given intravenous resuscitation via 0.9% sodium chloride (20 ml/kg), and ceftriaxone (80 mg/kg per day). Notably, two samples of sputum cultures at 24 h both confirmed *Streptococcus pneumoniae*. Delayed urinalysis showed 4+ proteinuria by dipstick. Combined with hypoalbuminemia, an unexpected diagnosis of NS was brought to mind irrespective of its absence in symptoms. Despite being hemodynamically stable and absent from fever, his dyspnea and period of headache became progressively worsening. Respiratory distress with percutaneous oxygen saturation of 88% even via a face mask was noted on hospital day 3. Both meningeal irritation and pathological signs were negative. Based on a high degree of suspicion for NS and increased vigilance of consequent hypercoagulability, the possiblity of thromboembolism was taken into account, which might be attributable to dyspnea and headache. This speculation had been corroborated by subsequent amazing results of targeted tests for disclosing the nature of the pulmonary and neurological lesions. Specifically, a computed tomography angiography (CTA) of chest demonstrated multiple intraluminal filling defects in the branch pulmonary arteries of both lungs, suggestive of PE in bilateral pulmonary arteries (Figures 1A,C,E), whereas no evidence of DVT was identified in bilateral lower limb veins by ultrasound. Meanwhile, the head CT manifested high-density shadows in the tentorium cerebelli and venous sinus, with normal results of repeat CSF analysis. Based on the correlation between headache episode and possible intracranial clot, cranial magnetic resonance imaging (MRI) and MR venography (MRV) were subsequently performed. Notably, multiple signal abnormalities were found in superior sagittal sinus, inferior sagittal sinus, straight sinus, bilateral transversal sinuses, bilateral sigmoid sinuses, vein of Galen, and torcula (Figures 2A–D), consistent with the imaging features of CVST.

**Table 1:** Patient's laboratory findings on admission.

Renal/liver function		Coagulation		Blood gas		Electrolytes	
Creatinine (μmol/L)	54	PT (s)	12.2	PH	7.374	Sodium (mmol/L)	138
Urea nitrogen (mmol/L)	5.7	APTT (s)	35.2	PCO_2_ (mmHg)	31.8	Potassium (mmol/L)	4.5
AST (IU/L)	25	Fibrinogen (mg/dl)	553	PO_2_ (mmHg)	98.2	Calcium (mmol/L)	2.37
ALT (IU/L)	30	TT (s)	17.6	BE (mmol/L)	−2.8	Chloride (mmol/L)	102
LDH (IU/L)	226	DDI (μg/ml)	7.9	HCO_3_^−^(mmol/L)	22.4	Magnesium (mmol/L)	0.74
Albumin (g/dl)	2.4	FDP (μg/ml)	45.8	Lactate (mmol/L)	1.1	Phosphorus (mmol/L)	1.67
TB (μmol/L)	4.2	Antithromin III (%)	44	Glucose (mmol/L)	4.5	Serum iron (μmol/L)	20.6

AST, aspartate aminotrasferase; ALT, alanine aminotransferase; LDH, lactate dehydrogenase; TB, total bilirubin; PT, prothrombin time; APTT, activated partial thromboplastin time; TT, thrombin time; DDI, D-dimer; FDP, fibrinogen degradation products; BE, base excess.

The patient immediately received treatment with high flow nasal oxygen (HFNO) therapy, subcutaneous injection of low molecular weight heparin calcium (150 U/kg per day), and continuous dripping intravenous infusion of urokinase (an initial bolus of 4 × 10^3^ U/kg for 15 min followed by a maintenance dose of 100 × 10^3^ U/kg per day). Of great concern, it was crucial to further confirm the underlying disease contributing to the pathogenesis of CVST and concurrent PE. It was noteworthy that multiple urinalysis had uniformly displayed massive proteinuria without hematuria. 1+ hyaline casts along with granular casts were detected in the sediment under a microscope at low power field (×10). Increase levels of urine β2-microglobulin were found with values between 1.64 and 2.20 μg/ml (normal value <0.25 μg/ml). Furthermore, a 24-h urine examination revealed 12.5 g proteinuria with a proteinuria to serum albumin (2.0 g/dl) ratio of 6.25. Meanwhile, dyslipidemia (elevated total cholesterol of 11.3 mmol/L and low density lipoprotein of 9.0 mmol/L), and progressive abnormalities in the coagulation-fibrinolysis system (antithrombin III of 37%, FDP of 57.1 μg/ml and DDI of 19.2 μg/ml), were identified. Given negative history of Henoch-Schonlein purpura along with unremarkable findings of human immunodeficiency virus, hepatitis B surface antigen, hepatitis C virus, autoimmune antibody and abdominal CT scan, the diagnosis of primary NS was ultimately confirmed.

**Figure 3:**
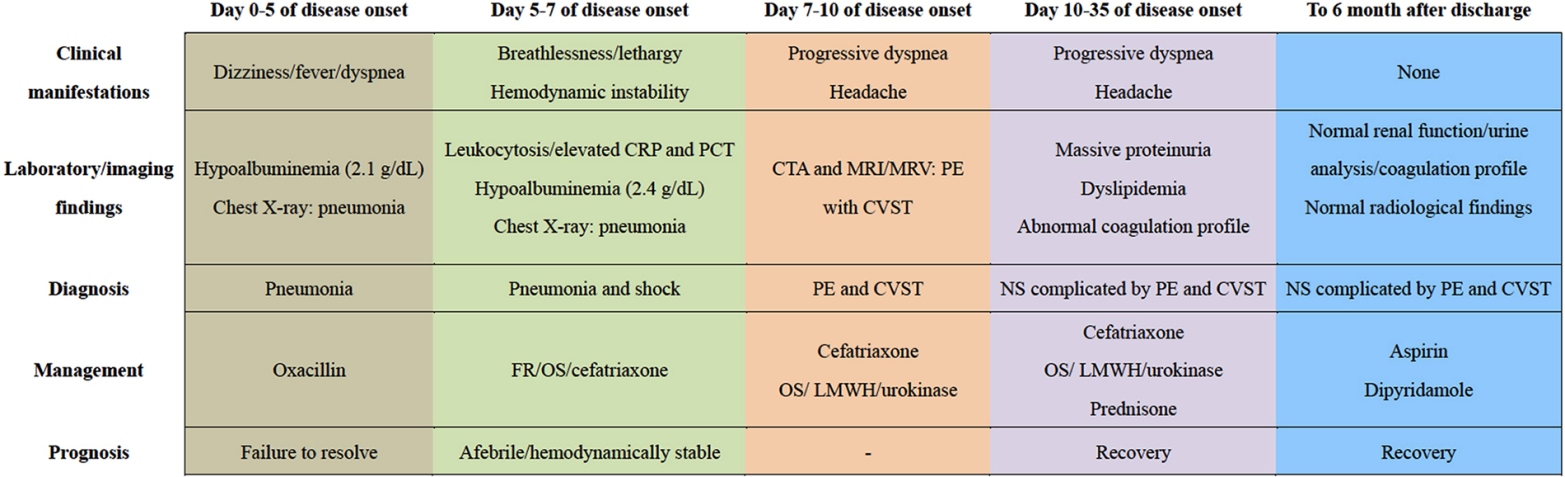
The timeline showcasing relevant data from the episode of care. CRP, C-reactive protein; PCT, procalcitonin; FR, fluid resuscitation; OS, oxygen supply; CTA, computed tomography angiography; MRI, magnetic resonance imaging; MRV, magnetic resonance venography; PE, pulmonary embolism; CVST, cerebral venous sinus thrombosis; LMWH, low molecular weight heparin; NS, nephrotic syndrome.

Given negative findings of T-spot assay, serum glucan/galactomannan tests, and radiographic evaluation suggestive of tuberculosis or fungal infections, additional therapy of oral prednisone (2 mg/kg per day) was administered for the treatment of NS. Despite this, screening for a comprehensive prothrombotic workup was also performed in order to exclude multiple additional risk factors for CVST and PE, including protein C/S, homocysteine, Leiden factor V, antineutrophil cytoplasmic antibodies (ANCA), anticardiolipin antibody, anti-Ro/SSA, anti-La/SSB, antiglomerular basement membrane (GBM) antibody, tumor marker concentrations, and bone marrow biopsy. All the results were within normal limits. Encouragingly, his headache and breathlessness gradually resolved. Radiographic recanalization of the infarcted pulmonary and cerebral vessels was identified on the 6th day (Figures 1B,D,F) and 20th day (Figures 2E–H) of antithrombotic therapy, respectively. Moreover, renal remission was achieved on the 13th day of steroid treatment, associated with complete resolution of the coagulation profile. The patient was discharged on oral prednisone, aspirin and dipyridamole after spending 28 days in hospital. Apart from normal renal function without proteinuria, no thromboembolic complications, neurological sequelae and side effects of drugs developed during follow-up for 6 months. Figure 3 summarized the Timeline with relevant data from the episode of care in this case.

## Discussion

CVST is one of the the most lethal form of VTE, leading to cerebral edema and, in some cases, infarction, haemorrhage and even herniation due to a large parenchymal lesion (19). On account of its vague nature of clinical presentations, ranging from asymptomatic cases to serious disease with impairment of consciousness, the diagnosis of CVST is probably delayed, masked and erroneously assumed to be related to other medical conditions that often coincide with CVST, particularly in childhood cases without apparent neurological deficits. Despite a well-established risk factor for VTE, NS remains to be a rare underlying etiology of CVST. In most cases, the first episode of VTE developed late in the course of the disease. It is extremely unusual that CVST occur initially at NS onset, specifically in combination with PE. Moreover, it is worthwhile to mention that because some cases with NS can be asymptomatic without edema and identified through chance proteinuria. This probably contributes to delayed or missed diagnosis of NS as well as the corresponding thromboembolic complications. Herein, we described a rare pediatric case who presented with CVST complicated by PE as the early onset of clinical manifestations induced by asymptomatic NS. This report highlighted that a high index of suspicion and prompt availability of neuroimaging assessment are clinically significant for the timely detection of affected individuals, particularly when patients are not improving on therapy in the setting of conditions known to predisposition of thromboembolic events.

Since approximately 95% of pediatric patients are triggered by at least one risk factor of prothrombotic conditions (6, 20), clinical awareness and acumen of the potential underlying disorders is the crucial point in early diagnosis of CVST and prevention of its short and long-term sequelae. However, current literature includes limited evidence on an association between CSVT and the risk factors, especially in the pediatric population. Till now, Sellers et al. have reported the largest case-control study of pediatric CVST patients, including 60 cases and 120 controls. In that study, head/neck infections (OR, 13.8; 95% CI: 4.87–38.7; *P* < 0.01), head/neck trauma (OR, 12.7; 95% CI, 2.88–56.2; *P* < 0.01), mechanical ventilation (OR, 9.32; 95% CI, 2.35–36.9; *P* = 0.01) were identified as independent risk factors for the development of CSVT (6). A relatively large retrospective study with 53 patients found that infections (43.8%) (including otomastoiditis, meningitis and sepsis), and inherited thrombophilia (26.4%) such as protein C/S deficiency, were the most frequent risk factors (7). Another study with 29 cases by Kar et al. reported infections (24.1%), mostly head/neck infections; inherited thrombophilia (44.8%) and a central venous catheter (6.9%) to be the dominant independent risk factors (9). In addition, chronic underlying disease such as malignancies, autoimmune disease, anemia, and NS were also recognized as CSVT risk factors in children (4, 10). Although NS is a known risk factor for CVST, its rarity deserve the caution to exclude additional underlying conditions as previously mentioned. As for our case, exclusion of head/neck infection, meningitis, sepsis, inherited thrombophilia, malignancies and autoimmune disease was based on physical examinations combined with CSF analysis, blood culture, tests for prothrombotic workup, imaging findings, bone marrow biopsy, and autoimmune antibody. Because of being performed after the onset of headache, the presence of a central venous catheter did not seem to be the direct causes of CVST. Therefore, CVST was believed to be induced by NS in this case.

A majority of reported literature speaks about the delay and difficulty in the diagnosis of CVST in children (10, 20). A previous study showed that the median time from symptom onset to diagnosis was 7 days (21), whilst it reached up to 10 days in our case. The substantially delayed diagnosis would probably give rise to devastating consequence or even death in this patient associated with concomitant PE (19, 22). Based on a review of the literature and experience of us, a variety of factors may divert the diagnosis toward other concomitant conditions, resulting in a delayed diagnosis of CVST in NS patients. First, in view of the prevalence of VTE in NS, the most commonly encountered VTE is RVT (70%), followed by DVT, and PE; in contrast, CVST is never a common pattern of thrombotic event, which has been reported that only one case was identified among 700 cases of children with NS (23, 24). As such, lack of awareness about CVST among pediatrician is a factor to be considered due to its rarity. Second, it has been noted that 8.6% of children with an initial diagnosis of NS might be asymptomatic without edema and were accidentally confirmed by proteinuria through a urinary screening test (25). In this situation, it is probably too challenging to trigger a diagnosis of NS, consequently resulting in ignorance about the associated CVST. Moreover, in children, symptoms at onset are even more nonspecific than in adults, solely presented as a sudden, new, and worsening headache without apparent neurological deficits in approximately 32% of cases (26). This may overlap with concomitant conditions such as infections, and mislead the etiological diagnosis to these more common diseases, contributing to low suspicion of CVST. Lastly, given the gradual enlargement of thrombus, initial head CT may be normal in nearly 30% of patients (27), reflecting that it is unreasonable to rule out CVST with head CT alone. Unavailability of MRI/MRV at initial work-up might hinder prompt detection of CVST by neuroimaging. Regrettably, in our case, headache was never considered to be caused by CVST, owing to low awareness of this disorder as well as negative findings of initial head CT. Irrespective of pronounced hypoalbuminemia, NS was overlooked due to absence of edema and delayed urinalysis. Despite the rarity, NS should always be included in the differential diagnosis of risk factors for CVST, even in absence of edema.

NS is associated with a high incidence of thromboembolism, whereas, when present, only 5% of the cases manifest as the initial presentation of NS (15, 17). As for our case, the surprise lied in the rarity of extraordinary early-onset of thromboembolism. In our review of the literature, several clinical clues are pivotal for identifying NS patients who are at highest risk for future thromboembolism. Firstly, age is an important modifier of thromboembolic risk, Increasing age was found to positively correlate with thromboembolic events, with the univariate odds ratio (OR) rising by 1.16 for each additional year of age advancement. Thus, adolescent age is presumably at highest risk, comparing an OR of 8.59 for those aged >12 years at diagnosis versus children aged <12 years (28). Additionally, there is overwhelming evidence that the risk of VTE proportionally increases with the severity of proteinuria and hypoalbuminemia. Relative to patients with albumin ≥4.0 g/dl, those with albumin level <2.5 g/dl at presentation have an almost 3-fold risk of VTE, with further increase in relative risk corresponding to even lower levels of serum albumin (29). Indeed, the ratio of proteinuria to serum albumin is a more accurate predictor of VTE than serum albumin levels or the magnitude of proteinuria alone (hazard ratio, 5.6) (11). Furthermore, on account of interplay among infectious pathogens, the immune system and coagulation system, infection has been recognized as a contributor to thrombosis in childhood NS (30). Several cohort studies have suggested that infection-induced exposure of tissue factor derived from endothelial damage was closely linked to VTE (31, 32). Particular pathogens, for instance *Staphylococcus aureus* and *Streptococcus pneumoniae*, have been shown to lead to an increased risk of thrombosis in hospitalized children with NS (33). Lastly, intravascular volume depletion, increases blood viscosity and slow blood flow, conferring enhanced RBC aggregation and clot formation (34). Collectively, the presence of aforementioned predisposing factors may have heightened the risk of thromboembolic attack and accelerated the onset and progression of CVST and PE in our NS patient. early thromboprophylaxis and specific radiological assessment might be proposed to this subset of high-risk candidates.

## Conclusion

A persistent clinical suspicion of CVST should be borne in mind in patients with a sudden, new or worsening headache, specifically among those with prothrombotic conditions. NS should always be considered in the differential diagnosis of risk factors for CVST, even in absence of edema. Since CVST and PE can be present simultaneously at extraordinary early-onset of NS, early radiological diagnosis is clinically substantial to proper management and satisfactory long-term outcomes.

## Data Availability

The original contributions presented in the study are included in the article, further inquiries can be directed to the corresponding authors.
